# The right occipital lobe and poor insight in first-episode psychosis

**DOI:** 10.1371/journal.pone.0197715

**Published:** 2018-06-01

**Authors:** Diana Tordesillas-Gutierrez, Rosa Ayesa-Arriola, Manuel Delgado-Alvarado, Jennifer L. Robinson, Javier Lopez-Morinigo, Jesus Pujol, M. Encarnación Dominguez-Ballesteros, Anthony S. David, Benedicto Crespo-Facorro

**Affiliations:** 1 Neuroimaging Unit, Technological Facilities,Valdecilla Biomedical Research Institute IDIVAL, Santander, Cantabria, Spain; 2 CIBERSAM, Centro Investigación Biomédica en Red de Salud Mental, Santander, Spain; 3 Department of Psychiatry, University Hospital Marqués de Valdecilla, School of Medicine, University of Cantabria-IDIVAL, Santander, Spain; 4 Department of Psychology, Auburn University, Auburn, Alabama, United States of America; 5 Department of Electrical and Computer Engineering, Auburn University, Auburn University Magnetic Resonance Imaging Research Center, Auburn, Alabama, United States of America; 6 Department of Kinesiology, Auburn University, Auburn, Alabama, United States of America; 7 Department of Psychosis Studies, Institute of Psychiatry, Psychology and Neuroscience, King’s College London, London, United Kingdom; 8 MRI Research Unit, Hospital del Mar, Barcelona, Spain; 9 UGC Psychiatry, Hospital Virgen Macarena, Sevilla, Spain; Universite de Lausanne, SWITZERLAND

## Abstract

Lack of insight is a core feature of non-affective psychosis and has been associated with poorer outcomes. Brain abnormalities underlying lack of insight have been suggested, mostly in the frontal lobe, although previous research showed mixed results. We used a voxel-based morphometry (VBM) analysis in 108 first-episode non-affective psychosis patients to investigate the pattern of brain structural abnormalities related to lack of insight. In addition, 77 healthy volunteers were compared with the patients classified as having poor and good insight. The shortened version of the Scale to Assess Unawareness of Mental Disorder was used to evaluate insight. Patients with poor insight (n = 68) compared with patients with good insight (n = 40) showed a single significant cluster (*k*_*c*_ *= 5834; PcFWE = 0*.*001*) of reduced grey matter volume (GMV) in the right occipital lobe extending to its lateral and medial surfaces, the cuneus, and the middle temporal gyrus. In addition, GMV at this cluster showed a negative correlation with the score of the SUMD (r = -0.305; p = 0.001). When comparing patients with poor insight with healthy subjects overall reductions of GMV were found, mainly in frontal and occipital lobes. Hence, poor insight in non-affective psychosis seems to be associated with specific brain abnormalities in the right occipital and temporal cortical regions. Dysfunction in any combination of these areas may contribute to lack of insight in non-affective psychosis. Specifically, the ‘right’ hemisphere dysfunction underlying impaired insight in our sample is consistent with previously reported similarities between lack of insight in psychosis and anosognosia in neurological disorders.

## 1. Introduction

Lack of insight is considered to be a cardinal feature of psychoses[1, 2]. Even at the time of diagnosis, up to 50–80% of first-episode psychosis (FEP) patients show poor insight into having a mental illness, which remains in some cases after psychosis improvement [3–5]. Importantly, lack of insight has been linked with treatment non-adherence[6]and poor long-term outcomes [7].

Little is known about the mechanisms that underlie poor insight in non-affective psychosis. Similarities between lack of insight in schizophrenia and unawareness of neurological deficits, termed anosognosia, has prompted speculation that they might share a common mechanism, which might involve perception and attentional processes. Some authors have linked right hemisphere dysfunction with poor insight [8, 9] in both neurological and psychiatric disorders [1, 10, 11].It is well established that subjects suffering right perisylvian lesions as a consequence of stroke, for example, are frequently unaware of their left-sided paralysis [12]. Indeed Morgan et al., in one of the first studies using voxel-based morphometry (VBM) in a FEP sample found a cluster of reduced grey matter volume (GMV) in a right hemisphere posterior region which correlated with the inability to relabel psychotic symptoms as abnormal [11].

On the other hand, frontal lobe changes affecting the left or both hemispheres have been found in many neuroimaging studies on insight. Particularly, dorsolateral frontal GMV reduction has been suggested to be critically involved in insight [9, 10]. Furthermore, functional imaging studies showed that the neural network underlying insight involves several regions, encompassing the medial frontal (including cingulate), the parietal, and the temporal cortices [13–18].Recent VBM studies investigating patients with chronic schizophrenia have confirmed associations between GMV reductions and poor insight in several brain regions [19–21]. In addition, VBM studies in FEP patients have shown volume reductions in the bilateral superior frontal gyri, the right inferior frontal gyrus, the right inferior temporal gyrus, the left cerebellum, the left insula, the bilateral superior temporal gyri, the precentralgyrus, the bilateral posterior cingulate gyrus, and the right cuneus to be associated with poor insight [11, 22].On the other hand, McFarland et al.[23] found excess of GMV in relation to impaired insight in the caudate, the insula, the putamen, the thalamus, and the cerebellum in first-episode affective and non-affective psychosis patients.

Variability among studies may be due to the small size of the examined samples and/or their clinical heterogeneity. Besides, most of the previous studies just compare patients with healthy controls. Thus, using a large FEP sample, with more statistical power to compare patients with good and poor insight while avoiding the effect of some of the confounders associated with chronic illness, may help to clarify the role of cerebral structures specifically associated with lack of insight.

The aim of the present study was to investigate the pattern of brain structural abnormalities related to lack of insight into mental illness in a large homogeneous sample of first-episode non-affective psychosis patients. We hypothesized that psychosis patients unaware of having a mental illness would show reduced GMV in brain regions associated with self-awareness and, in line with the anosognosia theory, predominantly in the right hemisphere.

## 2. Methods

### 2.1. Study setting and financial support

The studied sample was extracted from a large epidemiological program on first-episode psychosis (PAFIP) at University Hospital Marques de Valdecilla (Santander, Spain). Completemethodological information of this program has been reported elsewhere [24]. Subjects included in the present investigation were part of an on-going longitudinal intervention with a three-year follow-up (clinical trial NCT02305823). The study was approved by the Cantabria ethics committee in accordance with the international standards for research ethics (Declaration of Helsinki, 1964)and written informed consent was obtained from all the patients. Capicity of consent was determined by the psychiatrist (BC-F) through clinical interview. When minors were included in PAFIP, parents/legal guardians signed a parental permission consent document.

### 2.2. Subjects

A total of 264 subjects were included in the PAFIP program from February 2001 to December 2007,The inclusion criteria were: (1) age 15–60 years; (2) residencein the catchment area; (3) experiencing a FEP; (4) meeting DSM-IV criteria for schizophrenia, schizophreniform disorder, brief psychotic disorder, or schizoaffective disorder(5) no prior treatment with antipsychotic drugsor, if previously treated, a total life time of adequate antipsychotic treatment of less than 6 weeks.Patients were excluded if they met DSM-IV criteria for (1) drug dependence (except nicotine dependence), (2) mental retardation, or (3) had a history of neurological disease or head injury. Confirmation of diagnoses were done by using the Structured Clinical Interview for DSM-IV (SCID-I) [25], which was administered by an independent psychiatrist 6 months after the initial contact.

Of the 264 patients who entered the program, 153 agreed to participate in the MRI study. Of those who took part in this investigation, 22 did not complete the scan and 3 were excluded because of poor quality data. Twenty subjects of age older than 40 were also excluded, resulting in 108 patients, who were included in the final analysis (see Flow-chart). These patients have been randomly assigned to receive treatment with olanzapine (n = 24), risperidone (n = 22), ziprasidone (n = 16), quetiapine (n = 16), aripiprazole (n = 13) or haloperidol (n = 17) as part of the larger clinical trial. Only two patients had been minimally treated prior to randomization to antipsychotic treatments (one with quetiapine and one with haloperidol). Patients had a baseline structural MRI as soon as they could tolerate the procedure following the initiation of treatment. The mean time between initiation of treatment and MRI was 4.5 weeks (±3.6 SD).

Healthy volunteers (n = 77) were also recruited from the same localarea through advertisements. Exclusion criteria were: (1) current or past history of psychiatric, neurological or general medical illnesses, including substance dependence and significant loss of consciousness, which was determined using an abbreviated version of the Comprehensive Assessment of Symptoms and History[26] (2) history of psychosis in first-degree relatives. The selection of healthy controls was performed in order to obtain similar distribution in age, gender, laterality index, drug history and years of education as the patient population.

### 2.3. Insight assessment

Insight was assessed in the FEP patientsat 6 weeks after entering the program with the abbreviated version of the Scale to Assess Unawareness of Mental Disorder (SUMD) [27].

The abbreviated version of SUMD in schizophrenia [28] is a valid and reliable instrument for measuring insight in patients with schizophrenia and may be used by clinicians to accurately assess insight in clinical settings [29]. A short form of a scale is frequently associated with better acceptability in clinical practices. The abbreviated version of the SUMD (9 items: SUMD1: Awareness of a mental disorder, SUMD2: Awareness of the consequences of a mental disorder, SUMD3: Awareness of the effects of drugs, SUMD4: Awareness of a hallucinatory experience, SUMD5: Awareness of delusional ideas, SUMD6: Awareness of disorganised thoughts, SUMD7: Awareness of blunted affect, SUMD8: Awareness of anhedonia, SUMD9: Awareness of lack of sociability) may appear to be more practical than the long version and could lead to the inclusion of insight assessments as a part of routine clinical practice to offer individualised care. Each item was encoded in the same way with respect to the following modalities: not applicable (response of ‘0’ or missing data), aware (response of ‘1’), slightly aware/unaware (response of ‘3’), and seriously unaware (response of ‘5’).

First dimension, and particularly SUMD1 (Awareness of a mental disorder), which correlation is 0.99 with Awareness of disease, was considered the most representative measure of clinical insight, and scores ≤ 1 are considered as good insight[28, 30, 31].

### 2.4. Clinical assessment

The Brief Psychiatric Rating Scale total[32], the Scale for the Assessment of Negative Symptoms (SANS)[33] and the Scale for the Assessment of Positive Symptoms (SAPS)[34] in their validated Spanish versions were used to assess clinical symptoms at baseline and at the end of weeks 1, 2, 3, 4, and 6 of antipsychotic treatment. The same trained psychiatrist (BC-F) completed the clinical evaluation of patients. Handedness was evaluated by the Edinburgh Inventory [35], right handednessbeingdefined as an Edinburgh laterality index higher than 0.6.

Duration of untreated illness was defined as the time from the first unspecific symptom related to psychosis (for such symptom to be considered, there should be no return to previous stable level of functioning) to the date of initiation of an adequate dose of antipsychotic drug taken regularly. Duration of untreated psychosis was defined as the time from the first continuous (present most of the time) psychotic symptom to initiation of adequate antipsychotic drug treatment. Duration of prodromal period was defined as the period from the first unspecific symptoms related to psychosis (as defined above) to the first continuous (present most of the time) psychotic symptom. These disease-related time intervals were retrospectively evaluated. Age of onset of psychosis was defined as the age of emergence of the first continuous (present most of the time) psychotic symptom.

### 2.5. MRI data acquisition and image processing

In the first 12 weeks after entering the program patients were offered an MRI scan. High-resolution three-dimensional (3D) T1-weighted images were acquired on a 1.5-T whole-body scanner (SIGNA, GE, Milwaukee, WS, USA) at the University Hospital Marques of Valdecilla, Santander, Spain. Three-dimensional T1-weighted images, using a spoiled gradient-recalled acquisition in the steady state (GRASS) (SPGR) sequence, were acquired in the coronal plane with the following parameters: TE = 5 msec, TR = 24 msec, NEX = 2, rotation angle = 45°, FOV = 26 x 19.5 cm, slice thickness = 1.5 mm and a matrix of 256 x 192.

Voxel Based Morphometry (VBM) [36] was performed using the VBM5 toolbox (http://dbm.neuro.uni-jena.de/vbm/download/), an extension of the SPM5 software package (Statistical Parametric Mapping, Wellcome Department of Imaging Neuroscience, London, UK). The VBM pre-processing included the following steps. First, inspection for scanner artifacts and gross abnormalities for each subject, then, images were segmented into grey matter (GM), white matter (WM) and cerebrospinal fluid. In order to improve the quality of segmentation a Hidden Markov Random Field (HMRF) model [37] was applied to the segmented tissue. Afterwards, GM and WM images were imported into the DARTEL toolbox to create a population template from the complete dataset using a high-dimensional diffeomorphic registration algorithm DARTEL [38]. The obtained deformation fields were applied to the GM images to register them to Montreal Neurological Institute (MNI) standard space, followed by modulation in order to assess GMV differences and smoothing with a 5mm FWHM Gaussian kernel (voxel size 1x1x1).

### 2.6. Statistical analysis

Processed images were analyzed within the framework of the General Linear Model. Several t-test analyses were performed to investigate GMV differences between healthy controls and both insight psychosis patients groups using pairwise contrasts. Age at scan, gender and total intracranial volume were entered as covariates of no interest in the statistical design in order to regress out possible effects of these parameters on between-group volume differences. First, a primary cluster-forming voxel-level threshold of *p*<0.01 (uncorrected) was applied. Then, a cluster-level inference strategy was employed by evaluating obtained clusters at a cluster-extent threshold of *p*<0.05 family-wise error (FWE) corrected. All clusters sizes were adjusted for smoothness non-uniformity by means of the VBM5 toolbox[39]. Anatomical regions covered by significant clusters were identified using automated anatomical labeling[40].Pearson’s chi-square for categorical data and Student’s t-tests for continuous variables were used to evaluate differences in sociodemographic characteristics between controls and patients. The Statistical Package for Social Science, version 19.0 (SPSS Inc.,Chicago, IL, USA), was used for these analysis.

## 3. Results

### 3.1. Subjects

First Episode Psychosis (FEP), 40 (37%) individuals presented good insight and 68 (63%) poor insight. The control group included 77 healthy volunteers. Demographic and clinical characteristics of both patientgroups and socio-demographically similar healthy control subjectsare summarized in Table 1. There were no statistically significant differences in relevant socio-demographic characteristics between groups (all *p*>0.06, cut off *p*>0.05).

**Table 1:** Socio-demographic and clinical characteristics of the study groups.

	GoodInsight (n = 40)	Poor Insight (n = 68)	HealthyVolunteers (n = 77)	Statistics (dof)
**Males, n (%)**	28 (70)	46 (67.6)	51 (66.2)	χ^2^(2) = 0.71: *p* = 0.92
**Age at MRI, mean (SD), years**	27.36 (5.58)	26.49 (5.22)	26.18 (5.76)	F(2) = 0.60: *p* = 0.55
**Handedness, right n (%)**	35 (87.5)	56 (86.2)	69(90.8)	χ^2^(2) = 0.77: *p* = 0.68
**Height, mean (SD), cm**^**1**^	170.63 (9.40)	169.59 (8.22)	172 (8.35)	F(2) = 1.48: *p* = 0.23
**Age at onset, mean (SD), years**	26.77 (5.81)	25.50 (4.96)	-	F(1) = 1.46: *p* = 0.23
**Interval inclusion-mri, mean (SD) weeks**	4.13 (3.09)	4.77 (3.62)	-	F(1) = 0.81: *p* = 0.37
**Low parental socioeconomic status, n (%)**^**2**^	17 (42.5)	39 (58.2)	32 (43.2)	χ^2^(2) = 3.92: *p* = 0.14
**Education, mean (SD), years**	10.63 (3.18)	9.56 (2.89)	10.51 (2.59)	F(2) = 2.62: *p* = 0.08
**Alcohol users, n (%)**^**3**^	27 (67.5)	45 (66.2)	48 (65.8)	χ^2^(2) = 0.04: *p* = 0.98
**Cannabis users, n (%)**^**4**^	20 (50.0)	41 (60.3)	30 (40.5)	χ^2^(2) = 5.53: *p* = 0.06
**Tobaccousers, n (%)**^**4**^	21 (52.5)	45 (66.2)	44 (59.5)	χ^2^ = 2.02: *p* = 0.36
**DUP, mean, (SD), months**^**5**^	6.06 (8.73)	9.63 (15.00)	-	F(1) = 1.79: *p* = 0.18
**DUI, mean, (SD), months**^**5**^	15.42 (17.52)	25.33 (31.26)	-	F(1) = 3.39: *p* = 0.07
**DDP, mean, (SD), months**^**5**^	9.36 (13.88)	15.70 (25.66)	-	F(1) = 2.08: *p* = 0.15
**Symptomatology mean, (SD) (total scores)**				
** Negativedimension**	4.83 (4.98)	4.51 (4.91)	-	F(1) = 0.10: *p* = 0.75
** SANS**	7.00 (4.81)	6.01 (5.20)	-	F(1) = 0.96: *p* = 0.33
** SAPS**	13.33 (4.15)	13.88 (4.40)	-	F(1) = 0.42: *p* = 0.52
** Positive dimension**	7.38 (2.35)	7.40 (2.34)	-	F(1) = 0.002: *p* = 0.96
** Disorganizeddimension**	5.95 (3.30)	6.49 (3.46)	-	F(1) = 0.62: *p* = 0.43

**Abbreviations:** DUP, duration of untreated psychosis; DUI, duration of untreated illness; DPP, duration of premorbid period; SANS,Scale for the Assessment of Negative Symptoms; SAPS, Scale for the Assessment of Positive Symptoms. Statistics: F test value of ANOVA; χ2: value of chi-square test, dof: degrees of freedom.

^1^Based in data from 100 first episode of psychosis patients and 68 healthy volunteers.

^2^Based in data from 100 first episode of psychosis patients and 69 healthy volunteers.

^3^Based in data from 101 first episode of psychosis patients and 67 healthy volunteers.

^4^Based in data from 101 first episode of psychosis patients and 68 healthy volunteers.

^5^Based in data from 40 patients with good insight and 67 with poor insight.

### 3.2.VBM analysis

Results regarding differences between healthy subjects and the FEP patients group have been reported elsewhere[41].

#### 3.2.1. Healthy controls versus patients with poor insight

The comparison between healthy controls (HC) (n = 77) and patients with poor illness insight (n = 68) (HC>poor insight) showed an extensive decrease in GMV in five clusters (Tables 2–6):

**Table 2:** SPM results; areas where patients with poor illness insight show less grey matter volume than healthy controls (HC > poor IMI).

Anatomicalregion	Left		Right	
	Extent	T	ExteExtent	T
	K(%clust;%reg)	Max; mean (SD)	K(%clust;%reg)	Max; mean (SD)
*Cluster 1*: *k*_*c*_ *= 42041; P*_*cFWE*_*< 0*.*001; Voxelmaximumx*,*y*,*z [mm]*: *−50*,*-76*,*23; P*_*FWE*_ *= 0*.*017*				
Calcarinefissure	5521 (13.13; 30.56)	4.59; 2.96 (0.42)	4041 (9.61; 27.14)	5.04; 3.01 (0.51)
Middle occipital gyrus	5411 (12.87; 20.68)	5.18; 2.88 (0.41)		
Lingual gyrus	4370 (10.39; 26.07)	4.86; 2.87 (0.39)	2175 (5.17; 11.82)	3.82; 2.69 (0.28)
CerebelumCrus I	4300 (10.23; 20.65)	3.86; 2.86 (0.35)		
CerebelumCrus II	1940 (4.61; 12.80)	3.50; 2.73 (0.25)		
Fusiformgyrus	1614 (3.84; 8.73)	3.55; 2.77 (0.29)	1379 (3.28; 6.85)	3.63; 2.72 (0.27)
Inferior temporal gyrus	1515 (3.60; 5.92)	3.76; 2.75 (0.29)		
Middle temporal gyrus	1498 (3.56; 3.79)	4.56; 2.75 (0.31)		
Cuneus	1126 (2.68; 9.22)	4.33; 2.87 (0.40)	436 (1.04; 3.83)	3.40; 2.77 (0.27)
Superior occipital gyrus	1086 (2.58; 9.94)	3.98; 2.76 (0.27)		
Cerebelum VI	1040 (2.47; 7.67)	3.66; 2.67 (0.24)	45 (0.11; 0.31)	2.77; 2.50 (0.11)
Inferior occipital gyrus	874 (2.08; 11.61)	3.65; 2.66 (0.23)		
Cerebelum VII	863 (2.05; 18.44)	3.42; 2.76 (0.27)		
Cerebelum VIII	716 (1.70; 4.74)	3.48; 2.70 (0.25)		
Angular gyrus	288 (0.69; 3.07)	5.25; 2.93 (0.58)		
Vermis IV-V	205 (0.49; 3.85)	3.75; 2.66 (0.26)		
Cerebelum IV-V	175 (0.42; 1.94)	3.87; 2.85 (0.37)	39 (0.09; 0.57)	3.17; 2.66 (0.24)
ParaHippocampalgyrus	135 (0.32; 1.73)	3.08; 2.63 (0.21)	4 (0.01; 0.04)	2.54; 2.45 (0.08)
Precuneus	9 (0.02; 0.03)	2.77; 2.57 (0.13)	37 (0.09; 0.14)	3.17; 2.58 (0.19)
Inferior parietal gyrus	25 (0.06; 0.13)	3.12; 2.65 (0.24)		
Vermis VI	18 (0.04; 0.61)	3.24; 2.69 (0.29)		

Clusters were characterized by their extent Kc and significancePcFWE,family-wise error corrected for cluster extent and smoothness non-stationary, as well as by the localization x,y,z [mm] and corrected PFWE value of the maximum voxel. For anatomical regions within clusters, the number of voxels K, the percentage of the cluster covered %clust, the percentage of the region covered by the cluster %reg.

**Table 3:** SPM results; areas where patients with poor illness insight show less grey matter volume than healthy controls. (HC > poor IMI).

Anatomicalregion	Left		Right	
	Extent	T	Extent	T
	K(%clust;%reg)	Max; mean (SD)	K(%clust;%reg)	Max; mean (SD)
*Cluster 2*: *k*_*c*_ *= 30893; P*_*cFWE*_*< 0*.*001; Voxelmaximumx*,*y*,*z [mm]*: *4*,*51*,*26; P*_*FWE*_ *= 0*.*042*				
Superior Medial frontal gyrus	8016 (25.95; 33.49)	4.76; 2.80 (0.35)	7784 (25.20; 45.60)	4.83; 2.78 (0.34)
Middle frontal gyrus			2135 (6.91; 5.23)	3.78; 2.79 (0.30)
Supplementary motor area	1899 (6.15; 11.06)	4.86; 2.83 (0.38)	1657 (5.36; 8.74)	4.58; 2.83 (0.42)
Anterior cingulum	436 (1.41; 3.89)	3.71; 2.86 (0.37)	1634 (5.29; 15.56)	3.82; 2.80 (0.34)
Superior frontal gyrus	863 (2.79; 3.00)	4.53; 2.79 (0.35)	1578 (5.11; 4.86)	4.59; 2.79 (0.30)
MiddlesectionCingullum	1303 (4.22; 7.39)	4.43; 2.85 (0.39)	435 (1.41; 2.80)	3.75; 2.83 (0.35)
Middleorbitofrontalgyrus			837 (2.71; 10.31)	3.50; 2.82 (0.29)
Medial orbitofrontalgyrus	750 (2.43; 13.04)	3.48; 2.75 (0.27)	555 (1.80; 8.10)	3.47; 2.78 (0.30)
Inferior orbitofrontalgyrus			539 (1.74; 3.95)	3.31; 2.62 (0.21)
Inferior frontal gyrus, triangular part			168 (0.54; 0.98)	3.51; 2.65 (0.22)

Clusters were characterized by their extent Kc and significancePcFWE,family-wise error corrected for cluster extent and smoothness non-stationary, as well as by the localization x,y,z [mm] and corrected PFWE value of the maximum voxel. For anatomical regions within clusters, the number of voxels K, the percentage of the cluster covered %clust, the percentage of the region covered by the cluster %reg.

**Table 4:** SPM results; areas where patients with poor illness insight show less grey matter volume than healthy controls(HC > poor IMI).

Anatomicalregion	Left		Right	
	Extent	T	Extent	T
	K(%clust;%reg)	Max; mean (SD)	K(%clust;%reg)	Max; mean (SD)
*Cluster 3*: *k*_*c*_ *= 16654; P*_*cFWE*_*< 0*.*001; Voxelmaximumx*,*y*,*z [mm]*: *30*,*-97*,*14; P*_*FWE*_ *= 0*.*624*				
Middle occipital gyrus			6117 (36.73; 36.45)	3.82; 2.80 (0.32)
Middle temporal gyrus			2727 (16.37; 7.73)	3.86; 2.82 (0.33)
Superior occipital gyrus			2237 (13.43; 19.79)	3.71; 2.74 (0.29)
Inferior temporal gyrus			2104 (12.63; 7.39)	3.50; 2.76 (0.30)
Inferior occipital gyrus			1596 (9.58; 20.17)	3.41; 2.74 (0.24)
Cuneus			599 (3.60; 5.26)	3.51; 2.69 (0.25)
Angular gyrus			199 (1.19; 1.42)	3.62; 2.78 (0.25)
Lingual gyrus			139 (0.83; 0.76)	3.32; 2.63 (0.23)
Fusiformgyrus			53 (0.32; 0.26)	3.06; 2.56 (0.19)
CerebelumCrus I			43 (0.26; 0.20)	3.19; 2.50 (0.15)
Calcarinefissure			40 (0.24; 0.27)	3.03; 2.72 (0.22)
Cerebelum VI			10 (0.06; 0.07)	3.28; 2.76 (0.32)

Clusters were characterized by their extent Kc and significancePcFWE,family-wise error corrected for cluster extent and smoothness non-stationary, as well as by the localization x,y,z [mm] and corrected PFWE value of the maximum voxel. For anatomical regions within clusters, the number of voxels K, the percentage of the cluster covered %clust, the percentage of the region covered by the cluster %reg.

**Table 5:** SPM results; areas where patients with poor illness insight show less grey matter volume than healthy controls (HC > poor IMI).

Anatomicalregion	Left		Right	
	Extent	T	Extent	T
	K(%clust;%reg)	Max; mean (SD)	K(%clust;%reg)	Max; mean (SD)
*Cluster 4*: *k*_*c*_ *= 3825; P*_*cFWE*_ *= 0*.*015; Voxelmaximumx*,*y*,*z [mm]*: *52*,*-37*,*4; P*_*FWE*_ *= 0*.*981*				
Superior temporal gyrus			2552 (66.71; 10.16)	3.76; 2.80 (0.27)
Middle temporal gyrus			1084 (28.34; 3.07)	3.41; 2.70 (0.26)
Rolandicoperculum			95 (2.48; 0.89)	3.67; 2.79 (0.28)
Supramarginal gyrus			48 (1.25; 0.30)	3.72; 2.93 (0.41)

Clusters were characterized by their extent Kc and significancePcFWE,family-wise error corrected for cluster extent and smoothness non-stationary, as well as by the localization x,y,z [mm] and corrected PFWE value of the maximum voxel. For anatomical regions within clusters, the number of voxels K, the percentage of the cluster covered %clust, the percentage of the region covered by the cluster %reg.

**Table 6:** SPM results; areas where patients with poor illness insight show less grey matter volume than healthy controls (HC > poor IMI).

Anatomicalregion	Left		Right	
	Extent	T	Extent	T
	K(%clust;%reg)	Max; mean (SD)	K(%clust;%reg)	Max; mean (SD)
*Cluster 5*: *k*_*c*_ *= 3101; P*_*cFWE*_ *= 0*.*041; Voxelmaximumx*,*y*,*z [mm8*: *-43*,*-6*,*-8; P*_*FWE*_ *= 0*.*999*				
Superior temporal gyrus	1101 (35.50; 5.99)	3.47; 2.72 (0.26)		
Heschlgyrus	747 (24.09; 41.50)	3.50; 2.85 (0.31)		
Insula	553 (17.83; 3.72)	3.41; 2.73 (0.27)		
Rolandicoperculum	295 (9.51; 3.72)	3.28; 2.70 (0.24)		
Amygdala	17 (0.55; 0.97)	2.75; 2.55 (0.11)		

Clusters were characterized by their extent Kc and significancePcFWE,family-wise error corrected for cluster extent and smoothness non-stationary, as well as by the localization x,y,z [mm] and corrected PFWE value of the maximum voxel. For anatomical regions within clusters, the number of voxels K, the percentage of the cluster covered %clust, the percentage of the region covered by the cluster %reg.

Cluster 1: *kc = 42041; PcFWE<0*.*001*. This large cluster showed bilateral reductions mainly in the occipital and temporal lobes and cerebellum also extended to the parietal lobe and parahippocampalgyrus (Table 2).

Cluster 2: *kc = 30893; PcFWE<0*.*001*. This cluster showed bilaterally reduction through the frontal lobe (more in right orbitofrontal cortex) and anterior and medial part of the cingulum (Table 3).

Cluster 3: *kc = 16654; PcFWE<0*.*001*. This cluster is located in the right hemisphere, mainly in the occipital lobe (inferior, middle and superior occipital gyri, Cuneus, lingual gyrus and Calcarine fissure) extending to the middle and inferior temporal gyrus, angular gyrus in the parietal lobe and cerebellum (Table 4).

Cluster 4: *kc = 3825; PcFW<0*.*001*. 95% of the fourth cluster was located in the right temporal lobe (middle and superior temporal gyri) and extended to the Rolandic operculum and supramarginalgyrus. (Table 5).

Cluster 5: *kc = 3101; PcFWE<0*.*014*. This cluster showed reductions in the left hemisphere, mainly in the temporal lobe (superior temporal and Heschgyrus), Rolandic operculum, insula and limbic lobe (amygdala and putamen)(Table 6).

#### 3.2.2. Healthy controls versus patients with good insight

Whole brain GMV differences between 40 patients with goodinsight and 77 HC were identified mainly within cerebellum (tonsil, tuber and culmen), left inferior temporal lobe and fusiform *(kc = 4039; PcFWE = 0*.*001*). At p <0.05 cluster-level corrected, onecluster was identified (Table 7 and S1 Fig). No GMV increases were observed when comparing both groups.

**Table 7:** SPM results of grey matter analysis between patients with good illness insight and healthy controls (HC> good IMI).

Anatomicalregion	Left		Right	
	Extent	T	Extent	T
	K(%clust;%reg)	Max; mean (SD)	K(%clust;%reg)	Max; mean (SD)
*Cluster 1*: *k*_*c*_ *= 4039; P*_*cFWE*_ *= 0*.*001; Voxelmaximumx*,*y*,*z [mm]*: *−51*,*-54*,*-34; P*_*FWE*_ *= 0*.*059*				
CerebelumCrus I	3061 (75.79; 14.70)	4.99; 3.31 (0.60)		
CerebelumCrus II	458 (11.34; 3.02)	4.06; 3.09 (0.46)		
Cerebelum VI	191 (4.73; 1.41)	3.18; 2.60 (0.18)		
CerebelumVIIb	131 (3.24; 2.80)	3.82; 2.89 (0.38)		
Inferior temporal Gyrus	50 (1.24; 0.20)	3.73; 2.95 (0.40)		
Cerebelum VIII	27 (0.67; 0.18)	2.94; 2.57 (0.15)		

Clusters were characterized by their extent Kc and significancePcFWE, family-wise error corrected for cluster extent and smoothness non-stationary, as well as by the localization x,y,z [mm] and corrected PFWE value of the maximum voxel. For anatomical regions within clusters, the number of voxels K, the percentage of the cluster covered %clust, the percentage of the region covered by the cluster %reg

#### 3.2.3. Patients with good versus poor insight

**Fig 1:**
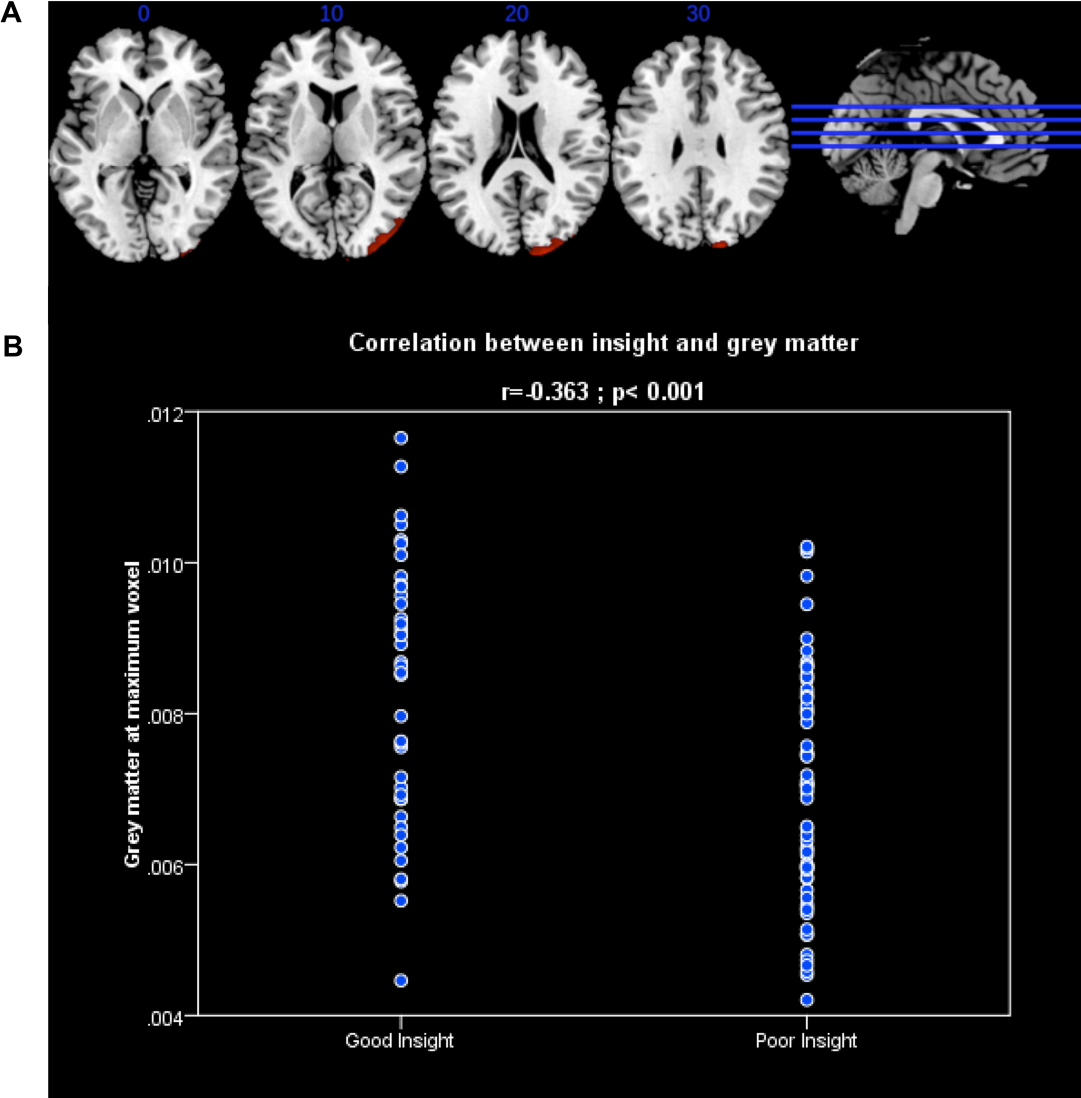
(A) In red are shown the results of the VBM contrast FEP patients with good insight vs. FEP with poor insight, cluster extends through the right middle and superior occipital gyri, cuneus and middle temporal gyrus. All results are in MNI space. (B) Correlation analysis: Grey matter values at maximum voxel of the contrast good insight > poor insight and insight.

The contrast revealed just a single significant cluster (*k*_*c*_ *= 5834; PcFWE = 0*.*001*) for smaller GMV in patients with poor insight (n = 68) compared to patients with good insight (n = 40). This was detected in the right occipital lobe, and extended to both its lateral and medial surfaces, and the cuneus. The cluster also extended into the middle temporal gyrus (BA 19 and BA 18) [x,y,z, coordinates 16, -95, 26] as shown in Table 8, Fig 1 and S2 Fig. In addition, we extracted grey matter voxels values at this cluster and using SPSS showed a negative correlation with the groups of insight (Pearson r = -0.363; p<0.001) (Fig 1).

**Table 8:** SPM results; areas where patients with poor illness insight show less grey matter volume than patients with good illness insight. (good IMI > poor IMI).

Anatomical region	Left		Right	
	Extent	T	Extent	T
	K(%clust;%reg)	Max; mean (SD)	K(%clust;%reg)	Max; mean (SD)
*Cluster 1*: *k*_*c*_ *= 5834; P*_*cFWE*_ *= 0*.*001; Voxel maximum x*,*y*,*z [mm]*: *16*,*-95*,*26; P*_*FWE*_ *= 0*.*306*				
Middle occipital gyrus			2945 (50.48; 17.55)	3.90; 2.87 (0.37)
Superior occipital gyrus			1661 (28.47; 14.69)	4.52; 3.19 (0.51)
Cuneus			466 (7.99; 4.09)	4.05; 2.79 (0.37)
Middle temporal gyrus			420 (7.20; 1.19)	3.24; 2.61 (0.18)

Clusters were characterized by their extent Kc and significancePcFWE, family-wise error corrected for cluster extent and smoothness non-stationary, as well as by the localization x,y,z [mm] and corrected PFWE value of the maximum voxel. For anatomical regions within clusters, the number of voxels K, the percentage of the cluster covered %clust, the percentage of the region covered by the cluster %reg.

## 4. Discussion

In the present work the neuroanatomical substrate of poor insight has been studied in a large epidemiological sample of FEP patients. Interestingly, we found a reduction in GMV in the lateral and medial gyrus of the right occipital lobe, the cuneus, and the middle temporal gyrus in patients with poor insight into having a mental illness compared to those with good insight.

The present results partially agree with some previous neuroimaging studies of insight in FEP subjects [11, 20, 22, 41, 42]. It has been previously shown that insight correlated with GMV in right frontal superior and inferior gyri, right frontal inferior operculargyrus, and right inferior temporal gyrus[22]. Symptom relabeling, the ability to identify and attribute the symptoms of psychosis as pathological, has been correlated with GMV decreases in right superior temporal and precentralgyri and right cuneus, and precuneus[11]. Similarly, Cooke at al.[20] found that right superior temporal gyrus GMV had a positive correlation with the ability to recognize experiences as abnormal. In addition, using surface-based analyses, cortical thickness of the inferior occipital gyrus was negatively correlated with mean awareness of illness [42]. Decreases of GMV in other areas such as the cerebellum, the left frontal and temporal cortices and the bilateral posterior cingulate and precuneus have also been related with poor insight [11, 20, 22]. Nonetheless, it seems that there is a predominance of abnormalities in right-sided areas.

Frontal lobe abnormalities have been one of the most consistent findings in the literature regarding insight in psychosis. In fact, structural changes in several areas of the frontal lobe have been linked to poor insight, particularly those affecting the prefrontal cortex [9, 22, 42–45]. And, indeed, frontal lobe mediated cognitive functions have been consistently associated with insight in patients with schizophrenia [8, 46–49]. Prefrontal cortex has been suggested to mediate insight in psychosis through impairment of a meta-representation of the self or deficits in Theory of Mind [50]. In this regard, medial prefrontal cortex has been consistently associated not only with the attribution of mental states to others but also to that of oneself [51]. However, most of the studies addressing insight in non-affective psychosis compared patients in the chronic phase of schizophrenia with healthy volunteers, which might have introduced the effect of the illness as a confounding factor. For example, a recent study found that schizophrenia patients with poor insight had widespread reductions in GMV as compared to those with preserved insight [52] but this may have been confounded by long-term exposure to neuroleptic drugs[53]. In addition, many of the previous studies adopted a region of interest approach rather than the less constrained whole brain approach. By contrast, our results suggest that differences in GMV due to different insight status are present at the earliest stage of the disease. Using VBM we have shown reductions in GMV in several areas of the frontal lobe when comparing patients with poor insight with healthy controls, but our findings also showed reductions in the temporal and occipital lobes in patients with poor insight compared to those with good insight. Taken together, it could be interpreted that reductions in frontal grey matter may be linked with the illness per se, while temporal and occipital reductions could be more specifically related to lack of insight into psychological (in addition to physical) change.

The right hemisphere predominance of our findings might be explained by the parallelism between insight and anosognosia for left-sided hemiplegia. Patients showing left hemiplegia, left spatial neglect, and anosognosia in comparison with those with hemiplegia and neglect but not anosognosia have lesions specifically associated with anosognosia distributed in right Brodmann’s premotor areas 6 and 44, right motor area 4, and the right somatosensory cortex, and also, although less frequently, in right prefrontal areas such as area 46 and the insula [54]. Indeed, several studies have associated anosognosia with damage of the right hemisphere motor and sensory cortices, the inferior frontal cortex, the insula, and the superior temporal gyrus[55, 56]. It is interesting to note that impaired self-awareness of motor symptoms in Parkinson’s disease patients has also been recently associated with right hemisphere structures[57]. The involvement of right hemisphere structures in anosognosia is complex and it has been suggested that, in addition to playing a key role in integrating somatosensory representations of the current state with an expected healthy state, the right hemisphere network plays a part in comparing a broader set of representations that include psychological and social skills.

The role of medial temporal gyrus in insight deserves comment. Previous studies have shown reductions in this area in patients with poor insight[52] or have correlated its volume with insight in patients with schizophrenia[20, 22]. This part of the temporal lobe has been classically associated with semantic and memory processing [58], and multimodal sensory integration [59]. Of note, it has been suggested that the medial temporal lobe plays a key role encoding episodic experiences during memory formation. Thus, difficulties in this function might hamper incoming corrective information and thus impair the updating of irrational beliefs [60]. In fact, memory impairment has been associated with poor insight in schizophrenia[61].

Finally, our findings of decreased GMV in the cuneus and medial gyrus of the right occipital lobe, are in keeping with two previous studies. One of them showed reduced GMV at the inferior occipital gyrus in patients with poor insight as compared to those with preserved insight [52], while another found an association between poor insight and volume reduction in a cluster extending posteriorly from the precuneus through the cuneus to the medial occipital gyrus[11]. The role of occipital structures in insight has been scarcely considered. Interestingly, Anton’s syndrome, visual anosognosia or denial of loss of vision in the setting of cortical blindness, is derived from bilateral occipital brain damage [62]. Moreover, patients with homonymous hemianopia due to unilateral occipital infarcts, may also be unaware of their visual defect [63]. Thus, occipital cortex might be involved in self-awareness, either in isolation such as in neurological conditions, or in coordination with other right-sided cortical areas in schizophrenia patients. However, further research is warranted to clarify the involvement of this and other herein discussed areas in insight in psychosis.

The main strength of this study lies on the methodology used, particularly in the sample recruitment and design. To the best of our knowledge, this is the largest VBM study carried out with a FEP sample regarding insight. Of most importance, our approach comparing not only patients and healthy controls but also patients with good versus those with poor insight, has allowed us to exclude the overall effect of schizophrenia per se on GMV. However, some limitations should be considered. Although our patients were only treated for a short period of time (mean = 4.53 weeks), the effect of antipsychotic medication on GMV cannot be excluded. In addition, VBM methodology has its own limitations, mainly concerning spatial normalization, smoothing and template[64]. However, a cautious methodological choice of pre-processing parameters and statistical options should lead to more reliable VBM results [65].

In conclusion, lack of insight in non-affective psychosis is associated with specific brain anomalies in right occipital and temporal cortical regions. Consistent with anosognosia in neurological disorders, lack of insight does not seem to be caused by damage to a specific brain area. Rather, insight in psychosis appears to involve a wider brain network, which includes temporal and occipital, and probably the interactions between these areas. Further research is needed to clarify how these brain regions, the circuitries linking them, or a combination of both underlie lack of insight in psychosis.

## Supporting information

S1 Fig In blue GMV reduction in patients with good insight with respect to healthy controls is overlaid over the contrast HC GMV greater than first episode of psychosis patients in red. As it can be seen the cluster falls inside the difference between patients and healthy subjects (overlaid area is shown in purple).
(TIF)

S2 Fig In blue GMV reduction in patients with poor insight with respect patients with good insight is overlaid on the red contrast that shows GMV reduction in patients with poor insight with respect healthy subjects (overlaid area is shown in purple). (TIF)

## Abbreviations

FEP: first episode psychosis
GMV: grey matter volume
SUMD: Scale to Assess Unawareness of Mental Disorder
