# Supplementary figures and images for: The right occipital lobe and poor insight in first-episode psychosis
Source: PLoS One. 2018 Jun 1;13(6):e0197715. doi: 10.1371/journal.pone.0197715 (PMC5983855; doi:10.1371/journal.pone.0197715)

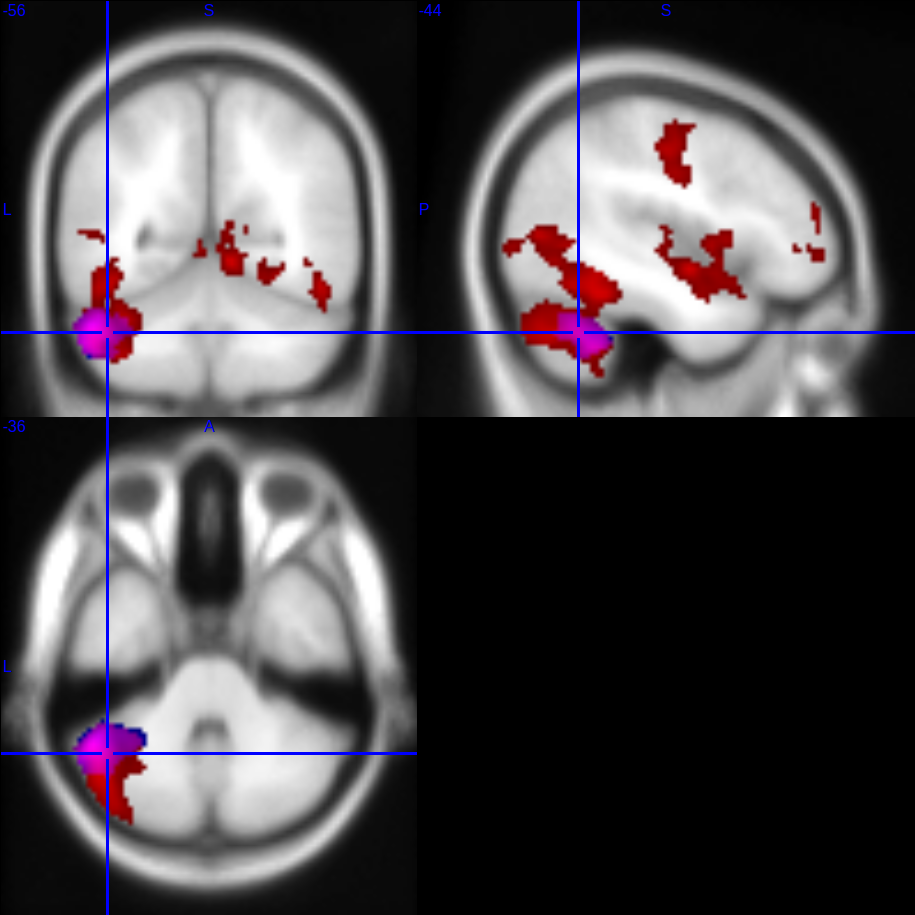

Supplement: S1 Fig — As it can be seen the cluster falls inside the difference between patients and healthy subjects (overlaid area is shown in purple). (TIF) [file pone.0197715.s001.tif]

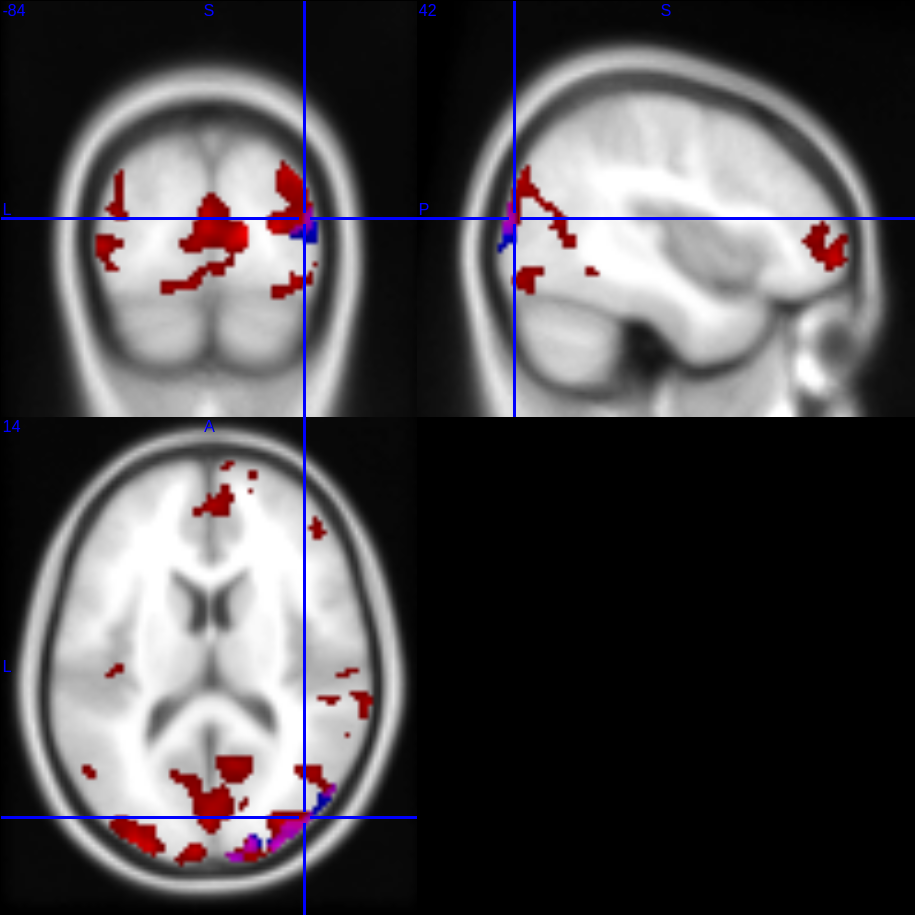

Supplement: S2 Fig — (TIF) [file pone.0197715.s002.tif]
